# A Compound AC1Q3QWB Selectively Disrupts HOTAIR-Mediated Recruitment of PRC2 and Enhances Cancer Therapy of DZNep

**DOI:** 10.7150/thno.35188

**Published:** 2019-06-24

**Authors:** Yansheng Li, Yu Ren, Yunfei Wang, Yanli Tan, Qixue Wang, Jinquan Cai, Junhu Zhou, Chao Yang, Kai Zhao, Kaikai Yi, Weili Jin, Lin Wang, Mingyang Liu, Jingxuan Yang, Min Li, Chunsheng Kang

**Affiliations:** 1 Department of Neurosurgery, Tianjin Medical University General Hospital, Tianjin 300052, China. Laboratory of Neuro-Oncology, Tianjin Neurological Institute, Department of Neurosurgery, Tianjin Medical University General Hospital and Key Laboratory of Neurotrauma, Variation, and Regeneration, Ministry of Education and Tianjin Municipal Government, Tianjin 300052, China.; 2 Department of Genetics, School of Basic Medical Sciences, Tianjin Medical University.; 3 Department of Pathology, Medical College of Hebei University, Baoding, Hebei 071000, China; 4 Department of Neurosurgery, the Second Affiliated Hospital of Harbin Medical University, Neuroscience Institute, Heilongjiang Academy of Medical Sciences, Harbin 150086, China.; 5 Department of Medicine, Department of Surgery, the University of Oklahoma Health Sciences Center, Oklahoma City, OK, 73104, USA.; 6 Affiliated Cancer Hospital & Institute of Guangzhou Medical University, Guangzhou510095, China.

**Keywords:** HOTAIR-EZH2, DZNep, APC2, β-catenin

## Abstract

Over 20% of cancer 'driver' genes encode chromatin regulators. Long noncoding RNAs (lincRNAs), which are dysregulated in various cancers, play a critical role in chromatin dynamics and gene regulation by interacting with key epigenetic regulators. It has been previously reported that the lincRNA HOTAIR mediates recruitment of polycomb repressive complex 2 (PRC2) leading to aberrant transcriptional silencing of tumor suppressor genes in glioma and breast cancer. Thus, lincRNA HOTAIR can serve as a promising therapeutic target. Herein, we identified a small-molecule compound AC1Q3QWB (AQB) as a selective and efficient disruptor of HOTAIR-EZH2 interaction, resulting in blocking of PRC2 recruitment and increasing tumor suppressors expression.

**Methods:** Molecular docking and high-throughput screening were performed to identify the small compound, AQB. RIP and ChIRP assays were carried to assess the selective interference of AQB with the HOTAIR-EZH2 interaction. The effects of AQB on tumor malignancy were evaluated in a variety of cancer cell lines and orthotopic breast cancer models. The combination therapy of AQB and 3-Deazaneplanocin A (DZNep), an inhibitor of the histone methyltransferase EZH2 was used *in vitro* and in orthotopic breast cancer and glioblastoma patient-derived xenograft (PDX) models.

**Results:** Tumor cells highly expressing HOTAIR and EZH2 were sensitive to AQB. APC2, as one of the target genes, was significantly up-regulated by AQB and led to degradation of β-catenin resulting in suppression of Wnt/β-catenin signaling which may contribute to inhibition of tumor growth and metastasis *in vitro* and in orthotopic breast cancer models. Remarkably, AQB enhanced the toxicity of DZNep *in vitro*. In orthotopic breast cancer and glioblastoma patient-derived xenografts (PDX) models, the combination of low doses of AQB and DZNep realized much better killing than DZNep treatment alone.

**Conclusion:** AQB is a HOTAIR-EZH2 inhibitor, which blocks PRC2 recruitment and has great potential as an effective agent for targeted cancer therapy.

## Introduction

Polycomb repressive complex 2 (PRC2)-mediated redistribution of H3K27me3, which aberrantly inactivates tumor suppressors, is a hallmark of facultative heterochromatin 1, 2. As the catalytic subunit of PRC2 and histone lysine methyltransferase (HMT) class enzymes, enhancer of zeste homolog 2 (EZH2) has been shown to be linked to poor prognosis and serves as a therapeutic target for various types of human cancers including breast cancer and glioma 3, 4. However, inhibitors of EZH2 have not provided clinical benefits and are limited to certain hematological malignancies. 3-Deazaneplanocin A (DZNep), an inhibitor of S-adenosy-L-homocysteine hydrolase that promotes degradation of the PRC2 complex, was demonstrated to be an anti-tumor agent in human acute myeloid leukemia and breast cancer 5, 6.

Recent evidence implicates that PRC2 depends on RNA guide to be deployed to thousands of mammalian genes 2, 7, 8. *In vitro* binding assays indicate that EZH2 is the high affinity RNA-binding subunit of PRC2 9, 10. To date, one of the most studied PRC2-interacting lincRNAs is HOTAIR. Initially, HOTAIR scaffold function was uncovered showing that its 5'domain bound the PRC2 whereas the 3'domain interacted with the LSD1/CoREST/ REST complex 11. Our previous study has demonstrated that in glioblastoma, HOTAIR regulates cell progression predominantly via the 5' domain-PRC2 axis, which is EZH2-dependent 12. Subsequently, EZH2-EED (embryonic ectoderm development protein, an essential subunit of PRC2) heterodimer was shown to be necessary and sufficient for binding to HOTAIR, and the minimal binding motif of HOTAIR was mapped to a folded 89-mer (212-300bp) domain 13. EZH2 is the subunit responsible for HOTAIR interaction, which protects HOTAIR from cleavage by RNase V1, while the EED subunit is required to stabilize the interaction 13. These findings offer opportunities to identify small molecules, which interfere with HOTAIR-EZH2 interaction, as a promising therapeutic option 13-17.

Here, we performed high-throughput screening 18 and identified a small-molecule compound, termed AC1Q3QWB (AQB), as a HOTAIR-EZH2 disruptor. AQB exhibited potent anti-tumor activity in the cells expressing high levels of HOTAIR and EZH2. We also showed that AQB significantly enhanced 3-Deazaneplanocin A (DZNep), an inhibitor of the histone methyltransferase EZH2, therapy in both orthotopic breast cancer and glioblastoma patient-derived xenograft (PDX) models. These data revealed that AQB is a HOTAIR-EZH2 inhibitor with high anti-tumor activity and thus has a great potential for therapeutic development.

## Materials and Methods

### Molecular modeling and docking

The process of in silico high-throughput screening was performed as described previously 18. The 3D structure of EZH2 (PDB ID: 4MI0) was from https://www.rcsb.org/. The HOTAIR (212-300bp) model was generated from the MC-Fold/MC-Sym program and analyzed for energy optimization using TINKER. Subsequently, we executed high-throughput molecular docking against the 1,990 NCI/diversity compounds employing the AutoDock program.

### Cells and drugs

The primary patient-derived glioblastoma cells, N5 and N33, were provided by Professor Fan (Beijing Key Laboratory of Gene Resource and Molecular Development, Laboratory of Neuroscience and Brain Development, Beijing Normal University) and were reported in our previous study 19. N5 and N33 cells were cultured in Dulbecco's Modified Eagle's Medium (DMEM_ / F12 (1:1) (Gibco) containing 10% fetal bovine serum (FBS). Cal51 breast cancer cells, SGC-7901 and MGC-803 gastric cancer cells were purchased from the China Academia Sinica Cell Repository (Shanghai, PR China); other cell lines were purchased from American Type Culture Collection (ATCC). All cells were incubated at 37 °C and 5% CO_2_. AQB was synthesized by WuXi AppTec Company. DZNep was purchased from Selleck. Cells were seeded the day before drug treatment.

### Samples for RNA sequencing and microarray data

Gene expression datasets and associated clinical data were downloaded from the following websites: TCGA (https://xenabrowser.net/hub/), and CGGA (http://www.cgga.org.cn).

### Cell viability, clonogenic, and Transwell assays, and flow cytometry

The Cell Counting Kit-8 (CCK8) assay (Dojindo, Japan) was employed to evaluate cell viability. A total of 2×10^3^ cells per well were seeded in 96-well plates on the day before treatment. After 48 hours of treatment, CCK8 was added and incubated for one hour before detection by Microplate Reader. The half inhibitory concentration (IC50) values were calculated by GraphPad Prism 6. For the clonogenic assay, cells were seeded at 300 cells per well in 6-well plates and cultured for 12 days. The colonies were fixed with 4% paraformaldehyde and stained with crystal violet. Transwell assay was performed using Transwell membranes without Matrigel. A total of 1×10^5^ cells in serum-free DMEM were added in the chambers, which were placed in 12-well plates containing 10% FBS. After treatment and incubation, the invading cells on the inserts were stained with crystal violet. The apoptosis detection was performed using the FITC Annexin V Apoptosis Detection Kit I (BD Biosciences). The cell cycle was assessed using the Cell Cycle and Apoptosis Analysis Kit (Beyotime). After staining, the cells were analyzed by flow cytometry.

### RNA-binding protein immunoprecipitation (RIP)

RIP experiment was performed using the Magna RIP RNA-Binding Protein Immunoprecipitation Kit (Merck Millipore) and ChIP-grade anti-EZH2 antibody (Cell Signaling Technology). RNA was extracted and transcribed to cDNA with random primers. Primers of lincRNAs are listed in Supplementary Table S1.

### Chromatin isolation by RNA purification (ChIRP)

ChIRP was performed following the previously described protocol described by Chang 20. DNA probes against the HOTAIR full-length sequence, which were designed by http://www.singlemolecule fish.com, were biotinylated at the 3' end. The mock control was described in a previous reference 21. All sequences of biotin probes are listed in Supplementary Table S2. TRIzol reagent was used to extract RNA to confirm RNA enrichment. DNA samples were amplified for analysis by agarose gel electrophoresis. For protein elution, beads were resuspended in DNase buffer with a cocktail of 100 µg/ml RNase A, 0.1 Units/microliter RNase H and 100 U/ml DNase I at 37°C for 30 min. Western blot analysis was performed to analyze the proteins.

### Chromatin immunoprecipitation (ChIP)

ChIP experiments were performed using the Millipore Magna ChIP^TM^A/G kit (Catalog # 17-10085). The antibodies against H3K27me3, EZH2, H3, and RNA Pol II phosphor-Ser2 were purchased from Cell Signaling Technology (CST). The primers for HOXD10, PCDH10, and PCDHB5 14 and for SMN2 Exon-325 22 have been described. The APC2 primers for site1, site2 and site3 are listed in Supplementary Table S1.

### Western blotting and agarose gel electrophoresis

Western blotting was performed as previously described 18. The primary antibodies used were anti-EZH2, anti-β-catenin, anti-P-β-catenin (Ser675), anti-H3 (1:1000 CST), anti-PCDHB5, anti-PCDH10, anti-Vimentin, anti-GAPDH (1:500 Proteintech), anti-HOXD10, anti-ZEB1 (1:300 SANTA CRUZ), anti-APC2, and anti-Snail (1:1000 Abcam). For DNA electrophoresis, 10 ul of sample DNA was loaded into each well of a 2% agarose gel and electrophoresed at 80 V for half an hour. Subsequently, the gel was imaged by ultraviolet light.

### RNA extraction and real-time quantitative PCR (RT-qPCR)

Nuclear and cytoplasmic fractions were isolated using 0.5% NP-40 (Solarbio) and RNase inhibitor (Promega) and total RNA was extracted using TRIzol reagent (Sigma). Thereafter, the cDNA was synthesized using the Prime Script RT kit (Promega). The RT-qPCR was performed in triplicate using LightCycler 2.0 (BIO-RAD). The values were normalized to GAPDH for the total RNA and cytoplasmic RNA, and U6 was used as an endogenous control for nuclear RNA. PCR primers were designed on NCBI (http://www.ncbi.nlm.nih.gov/tools/prim er-blast/) and the primer sequences are listed in Supplementary Table S1.

### Immunofluorescence assays

The immunofluorescence assay was performed as previously described 19.

### siRNA transfection and lentiviral infection

The cells were plated in a six-well plate at 70%-80% confluence. The non-targeting siRNA scramble control and siRNA targeting EZH2 (Shanghai GenePharma) were transfected into N33 and MDA-MB-231 cells using Lipofectamine 3000 (Invitrogen). The cells were harvested for RT-qPCR and Western blot analyses after 48 hours. The sequences are provided in Supplementary Table S2. Lentiviruses containing the full HOTAIR sequence and a negative control sequence were obtained from GeneChem (Shanghai, China).

### Orthotopic nude mouse models and treatment

All experimental protocols were conducted according to Tianjin Medical University guidelines for animal research and were approved by the Institutional Animal Care and Use Committee.

MDA-MB-231 orthotopic breast cancer model was as previously described 18. Seven days after implantation, 50 µl AQB (50 mg/kg, dissolved in DMSO) or 50 µl DMSO was administered by intraperitoneal injection (n=5 per group) every two days for 40 days (Figure 5). For the combination treatment, 50 µl DMSO, 50 µl AQB (0.2 mg/kg), 50 µl DZNep (1 mg/kg), and 50 µl mix (0.2 mg/kg AQB and 0.2 mg/kg DZNep) were injected intraperitoneally (n=5 per group) every two days for 40 days (Figure 7). The tumor volume was calculated using a caliper by the following formula: volume=length*width^2^. On day 40, the mice were euthanized, tumors were removed and weighed, and mouse hearts, livers, spleens, lungs, and kidneys were removed for bioluminescence analysis.

For the glioblastoma PDX model, tumors were surgically resected from patients diagnosed with glioblastoma. Subsequently, tumor tissues were cut into 1 mm^3^ blocks and planted subcutaneously in female BALB/c-nu mice. Four weeks later, PDX models were successfully established, and the intracranial PDX models were surgically established from the subcutaneous PDX tumors. Seven days later, 50 µl DMSO, 50 µl AQB (0.2 mg/kg), 50 µl DZNep (1 mg/ kg), and 50 µl mix (0.2 mg/kg AQB and 0.2 mg/kg DZNep) were injected intraperitoneally (n=5 per group) every two days until the mice were euthanized.

### Statistical analysis

Statistical significance was determined using two-tailed Student's *t*-test or ANOVA for functional analysis. Survival curves were plotted using Kaplan-Meier survival plots, and a log-rank test was used to test significance. The correlation of samples was examined using two-sided Pearson correlation. All statistical analyses were performed using SPSS 22.0 software and GraphPad Prism 6. The error bars in the figures represent the mean ± s. d. from at least three independent experiments. P<0.05 was considered statistically significant.

## Results

### Tumor cells with high levels of HOTAIR and EZH2 are sensitive to AQB

**Figure 1:**
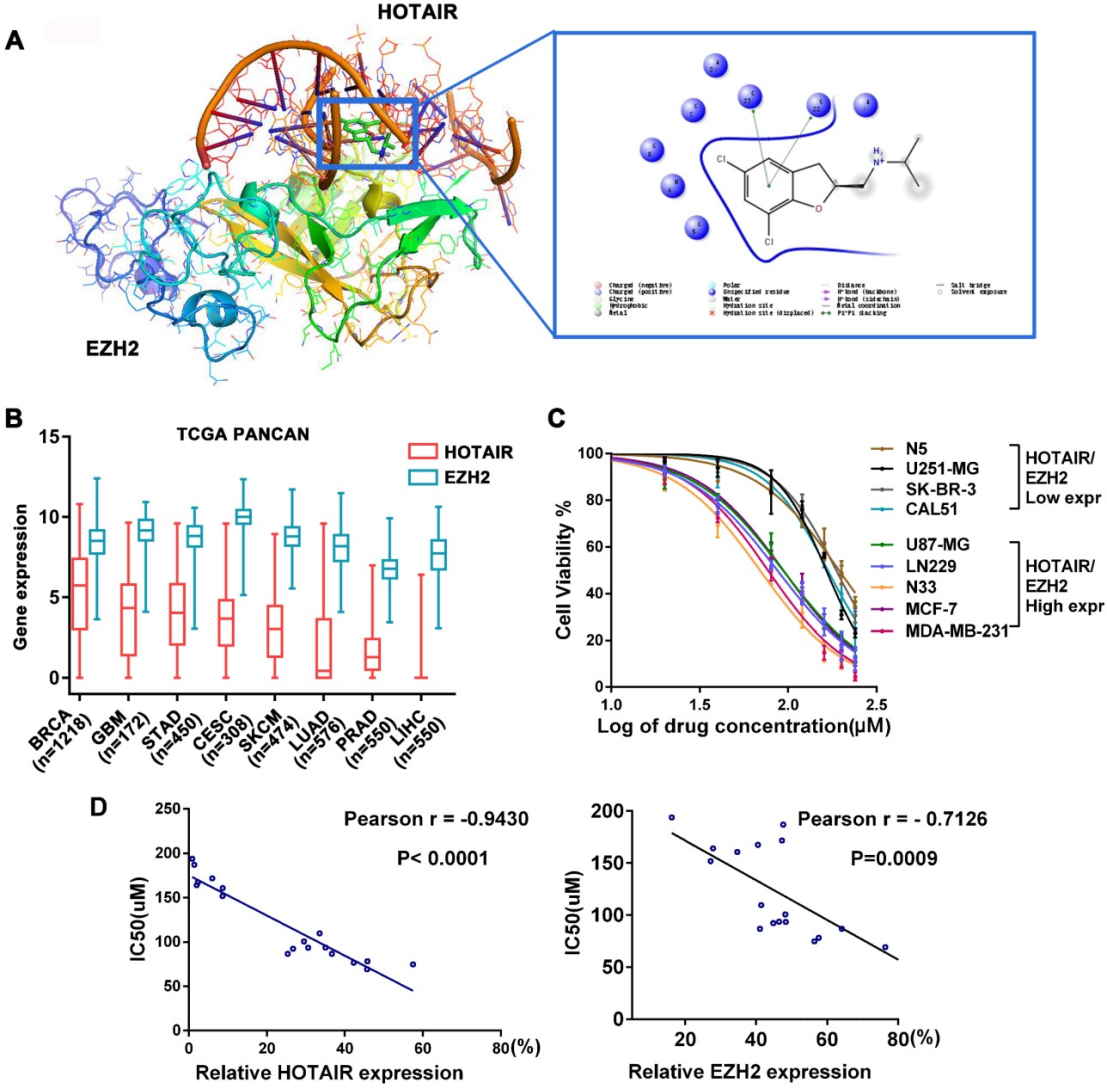
** AQB has low IC50 in cancer cells expressing high levels of HOTAIR and EZH2.** A) The docking results of AQB, HOTAIR, and EZH2. One nitrobenzene fragment of AQB formed π-π stacking with the 5'functional domain of HOTAIR. B) Box plot of 8 high-expressing HOTAIR and EZH2 tumors from TCGA Pan-Cancer Atlas Hub. C) Cell viability assay for glioblastoma and breast cancer cell lines after treatment with AQB for 2 days. Bars represent 3 independent experiments. D) The correlation analysis between the IC50 and levels of HOTAIR (left) or EZH2 (right) in 18 cell lines.

Frequent aberrant expression of HOTAIR and EZH2 has been detected in a wide range of tumors. Hence, the HOTAIR-EZH2 complex has been well established as a critical therapeutic target 13-17, 23. To identify a small molecule that disrupts HOTAIR-EZH2 interaction, we carried out *in silico* high-throughput screening based on the 3D structures of EZH2 and the 5'domain of HOTAIR. We found that a small compound, named AQB, was located between HOTAIR and EZH2 through intramolecular π-π stacking (Figure 1A). Furthermore, AQB was easy to synthesize (Figure S1A) and can be readily adapted for large-scale manufacturing.

We next analyzed the PanCancer data of TCGA and identified eight tumors with high expression of HOTAIR and EZH2. Among the eight tumors, the invasive breast cancer (BRCA) and glioblastoma (GBM) were the top two that highly expressed HOTAIR. (Figure 1B). We also examined background levels of HOTAIR and EZH2 in 18 cell lines of the 8 tumors and compared with HEK293 cells (Figure S1B). Next, we tested the toxicity of AQB in breast cancer, glioblastoma (Figure 1C) and 6 other tumor cell lines (Figure S1C). We found that cell lines with high levels of HOTAIR and EZH2 had lower IC50 for AQB (Figure 1C and Figure S1D). Correlation analysis showed a significant inverse correlation between AQB IC50 and HOTAIR or EZH2 levels (Figure 1D). These results indicated that cancer cells with high levels of HOTAIR and EZH2 are sensitive to AQB.

We further analyzed the subtypes of glioma and breast cancer. Interestingly, we found that HOTAIR and EZH2 were highly co-expressed in TERT mutations only subtype of glioma and triple-negative subtype of breast cancer (Figure S2A-D). Furthermore, correlation analysis in TCGA glioblastoma and breast cancer datasets showed a positive correlation with the expression of HOTAIR and EZH2. (Figure S2E). These findings implied that AQB may benefit patients whose tumors co-express high levels of HOTAIR and EZH2.

### AQB selectively blocks HOTAIR-EZH2 interaction and reduces recruitment of PRC2

**Figure 2:**
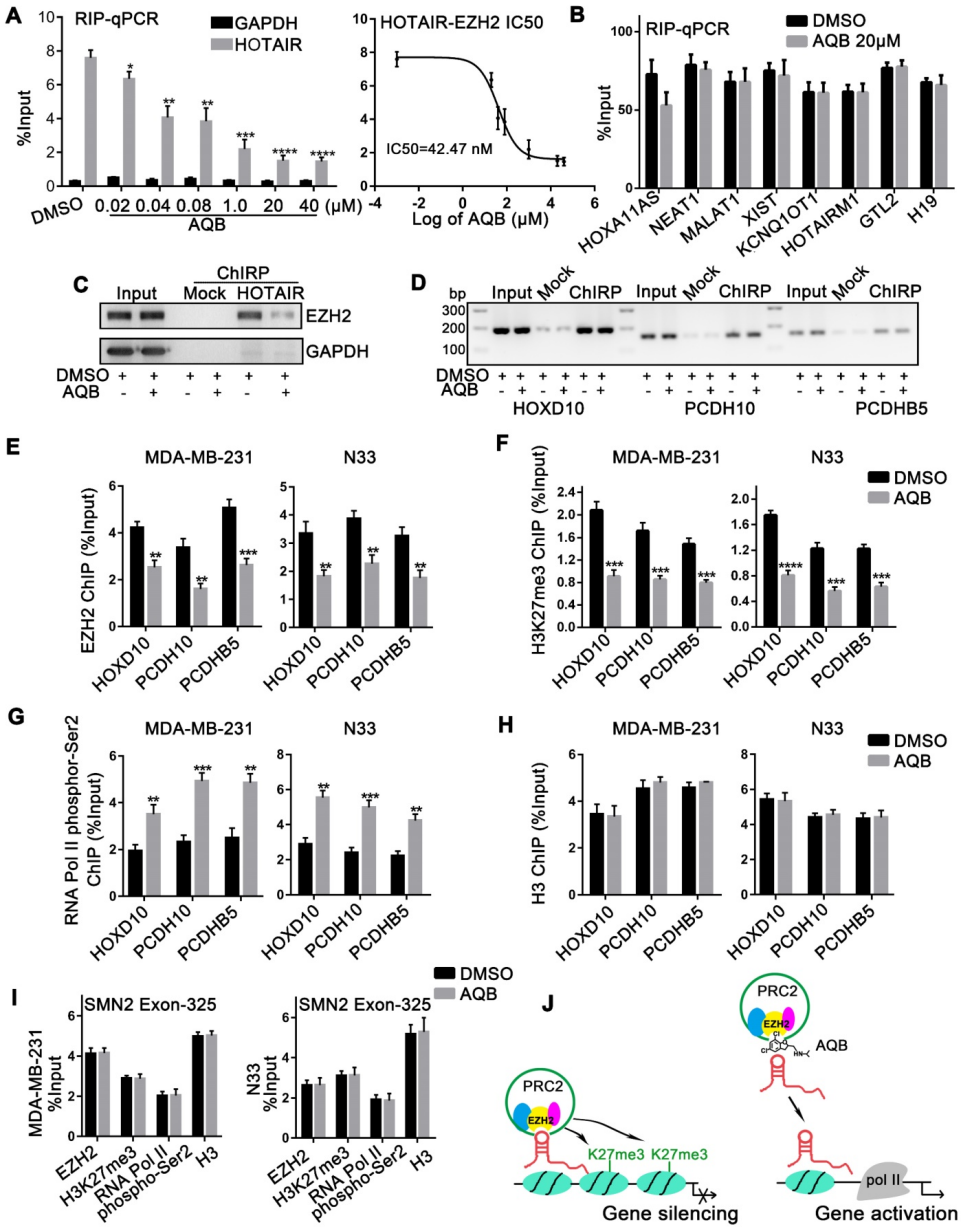
** AQB selectively blocks the HOTAIR-EZH2 interaction and weakens the recruitment of PRC2.** A) Following treatment with AQB at indicated doses, retrieved HOTAIR from EZH2-RIP assay was determined by RT-qPCR in MDA-MB-231 cells. GAPDH was the negative control. The dose-response curve revealed the HOTAIR-EZH2 IC50. B) Levels of indicated EZH2-binding lincRNAs were determined by qPCR after AQB (20µM) treatment. C) ChIRP-Western blot confirmed the AQB disruption of HOTAIR-EZH2 interaction. The protein fraction from Input, Mock-ChIRP and HOTAIR-ChIRP samples were analyzed using antibodies against EZH2 and GAPDH. HOTAIR ChIRP revealed less recovered protein in AQB treatment than DMSO treatment. GAPDH was not detected in any sample except Input. D) DNA fractions from each ChIRP sample were analyzed by 2% agarose gel electrophoresis after PCR and normalized to input. Mock ChIRP failed to retrieve substantial DNA from any locus. E-H) ChIP at the HOXD10, PCDH10, and PCDHB5 loci in N33 and MDA-MB-231 cells that were treated with 40nM AQB for 24 hours using antibodies against E) EZH2, F) H3K27me3, G) RNA polymerase II phospho-Ser2 and H) H3, I) ChIP at the promoter of SMN2, a PRC2-regulated gene, for EZH2, H3K27me3, RNA polymerase II phospho-Ser2, and H3, after treatment with 40nM AQB for 24 hours. J) Mechanism of action of AQB. Data are represented as mean ± s.d.; n = 3 independent experiments. ****P<0.00001, ***P<0.0001, **P<0.001, *P<0.05.

To further define the effect of AQB on HOTAIR and EZH2, we first examined whether AQB altered RNA and/or protein levels of HOTAIR or EZH2. The qPCR analyses revealed that neither total HOTAIR RNA nor cytoplasmic and nuclear HOTAIR RNA levels were significantly changed after AQB treatment in glioblastoma and breast cancer cells (Figure S3A, B). Also, total protein levels of EZH2, EED, and SUZ12 were not altered following AQB treatment (Figure S3C). However, we observed that the global level of H3K27me3 was decreased after 72 hours of AQB treatment (Figure S3D). Therefore, we proposed that AQB might block the interaction, and not the levels, of HOTAIR and EZH2. To further test this, we performed RIP in U87-MG and MDA-MB-231 cells after AQB treatment for 48 hours. We simultaneously examined other six lincRNAs that also bind to EZH2, including HOXA11AS 24, HOTAIRM1 25, NEAT1 19, MALAT1 26, XIST 27, and KCNQ1OT1 28. Interestingly, we found that only HOTAIR was reduced after AQB treatment, while other immunoprecipitated lincRNAs were unchanged (Figure S3E). This indicated that AQB selectively disrupted HOTAIR-EZH2 interaction. Given that the IC50 of AQB was about 80µM, the concentration at which it disrupted the HOTAIR-EZH2 interaction may be very low. Subsequently, we demonstrated that AQB was able to interfere with the HOTAIR-EZH2 interaction in a dose-dependent manner at a very low dose (IC50=42.47nM) (Figure**2**A). Other EZH2-binding lincRNAs, including GTL2 10, and H19 29, were not affected by AQB (Figure 2B).

To further verify the direct impact of AQB on HOTAIR-EZH2, we carried out ChIRP in MDA-MB- 231 cells treated with 40nM AQB. We observed that AQB significantly inhibited HOTAIR recruitment of EZH2 (Figure 2C). HOTAIR-associated genomic DNA purified by ChIRP was also examined using the promoter primers of HOTAIR-PRC2 target genes, including HOXD10, PCDH10, and PCDHB5 14. No significant alteration was detected after AQB treatment (Figure 2D), suggesting that AQB did not affect HOTAIR-chromatin interaction.

To demonstrate whether AQB blocked the recruitment of PRC2, we characterized the chromatin changes at HOXD10, PCDH10, and PCDHB5 loci in response to AQB treatment for 24 hours using ChIP. We observed a loss of EZH2 occupancy as well as decreased H3K27me3 at HOTAIR-PRC2 target loci (Figure 2E, F). Furthermore, RNA Pol II phospho-Ser2 (phosphorylation of serine 2 in RNA polymerase II) level was elevated indicating transcriptional elongation (Figure 2G). By contrast, the pan-H3 level was not affected by AQB treatment (Figure 2H). Also, no changes in PRC2 association were observed at another lincRNA SMN-AS1-mediated PRC2 target locus, SMN2 22, upon AQB treatment (Figure 2I). Taken together, these results suggested that HOTAIR- mediated PRC2 recruitment and histone methyltransferase activity at target gene loci can be selectively inhibited by the small-molecule compound AQB by mechanisms that may include steric blocking of the specific HOTAIR-EZH2 interaction or disruption of a secondary structure within HOTAIR recognized by EZH2 (Figure 2J).

### AQB leads to up-regulation of HOTAIR-PRC2 target genes

**Figure 3:**
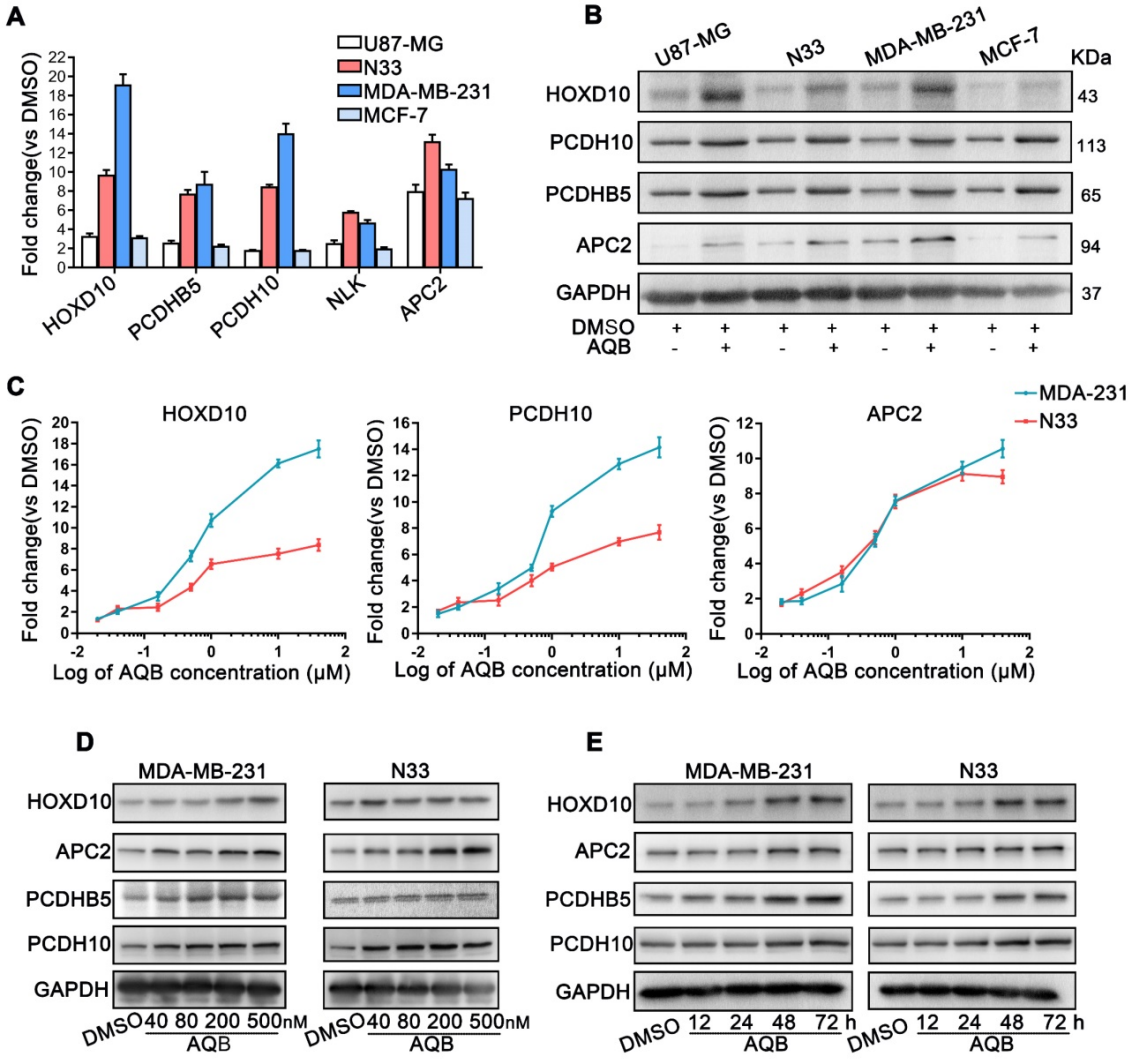
** AQB increases the expression of HOTAIR-PRC2 target genes.** A) Relative mRNA levels of the well-established HOTAIR-PRC2 target genes were measured by qPCR after treatment with 40µM AQB for 48 hours. B) Western blot analysis of indicated target genes after treating with 40µM AQB for 48 hours. C) Relative mRNA levels of target genes were measured by qPCR after treatment with indicated concentrations of AQB for 48 hours. D) Western blot analysis of target genes after treatment with indicated concentrations of AQB for 48 hours. E) Western blot analysis of target genes after treatment with 40nM AQB for indicated time periods. GAPDH was used as loading control. Data are represented as mean ± s.d.; n = 3 independent experiments.

Previous studies demonstrated that several genes were regulated by HOTAIR-mediated PRC2 silencing, including HOXD10, PCDH10, PCDHB5, APC2 14, and NLK 30. Therefore, we next tested whether AQB was able to up-regulate the mRNA and protein levels of these HOTAIR-PRC2 target genes. The results showed that mRNA and protein levels of these target genes, especially APC2, were significantly elevated in glioblastoma and breast cancer cell lines after treatment with 40µM AQB (Figure 3A, B). Considering the previous results that AQB was able to inhibit HOTAIR-EZH2 interaction at a very low dose (IC50=42.47nM), we further tested mRNA levels of the target genes in a set of concentration gradients of AQB from 20nM to 40µM. RT-qPCR analyses showed a concentration-dependent increase of HOXD10, PCDH10 and APC2 mRNA transcripts in N33 and MDA-MB-231 cells (Figure 3C). Furthermore, overall protein levels of the target genes also increased in a dose- and time-dependent manner (Figure 3D, E). These data suggested that low-dose AQB can effectively up-regulate both mRNA and protein levels of HOTAIR-PRC2 target genes.

### AQB suppresses Wnt/β-catenin signaling by upregulating APC2 expression

**Figure 4:**
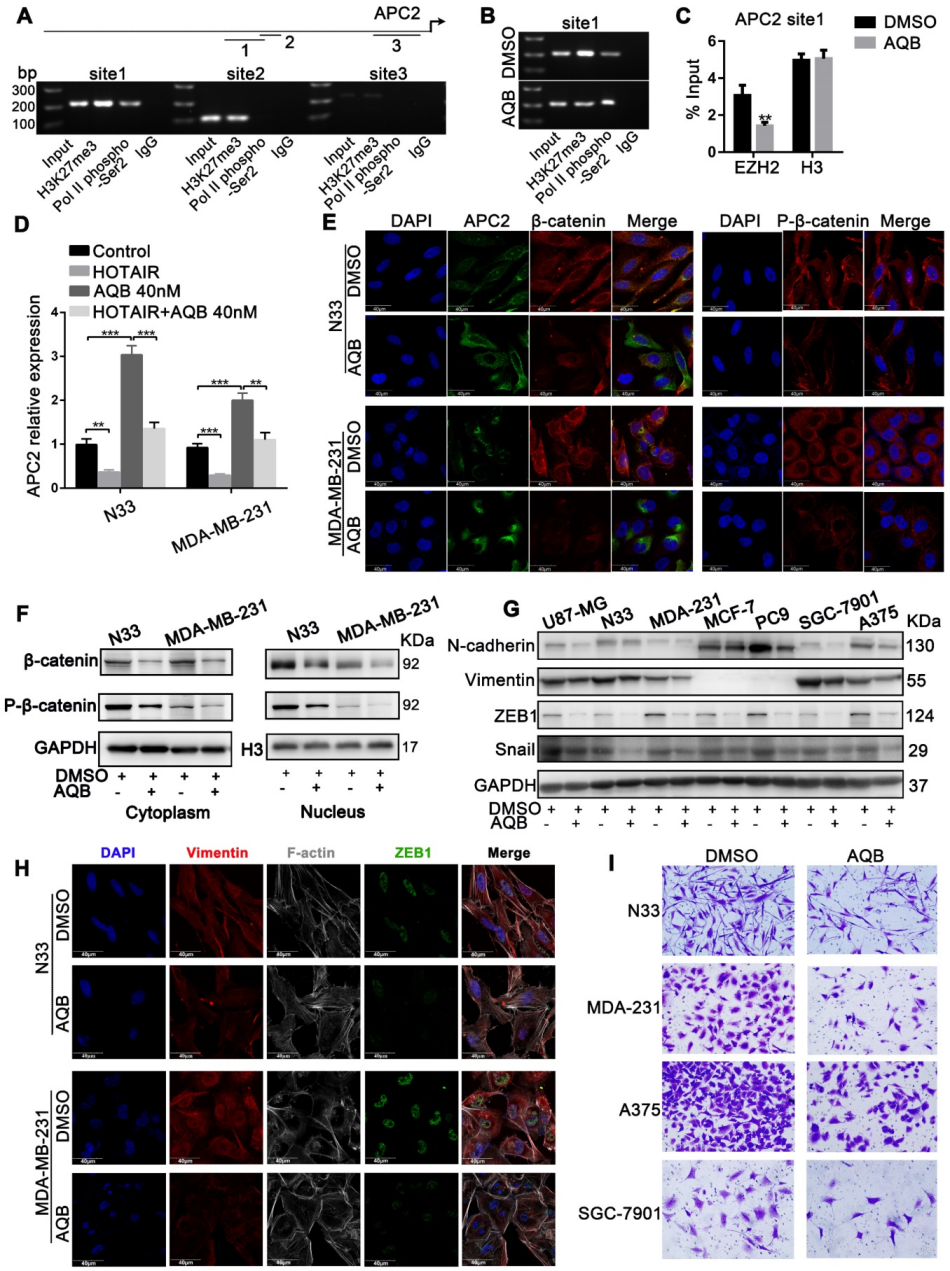
** APC2 is up-regulated by AQB, resulting in degradation of β-catenin and suppression of Wnt/β-catenin signaling.** A) The ChIP products were analyzed by agarose gel electrophoresis to determine the H3K27me3-binding sites. The long upper line represents the 2,000bp upstream of the transcription initiation site. The arrow indicates the direction of transcription. The three shorter lines represent the three predicted H3K27me3-binding sites. B) C) Following treatment with DMSO or 40nM AQB for 24 hours, H3K27me3, RNA pol II phospho-Ser2 ChIP, EZH2 and H3 ChIP assays were performed. D) RT-qPCR shows mRNA levels of APC2 in the cells transfected with HOTAIR or treated with AQB (40nM) or both. E) Immunofluorescence assay displays the APC2, β-catenin and p-β-catenin levels, as well as cellular distribution after the treatment with 200nM AQB for 48 hours. DAPI was used to stain the nuclei. Scale bar, 40μm. F) Western blot analysis of β-catenin and p-β-catenin levels in the nucleus and the cytosol lysates after AQB treatment. G) Western blot analysis of Wnt/β-catenin target genes and mesenchymal markers in a panel of cancer cell lines after treatment with AQB or DMSO. H) Confocal microscopy analysis of F-actin cytoskeleton, ZEB1 and Vimentin after treatment with AQB or DMSO. Bar, 40μm. I) Transwell assay shows inhibition of cell invasion by AQB in indicated cancer cell lines. Data are presented as mean ± s.d.; n = 3 independent experiments. ***P<0.0001, **P<0.0001.

As shown in Figure 3, among the HOTAIR-PRC2 target genes, AQB significantly induced the up-regulation of APC2. We further characterized APC2 and associated signaling alterations upon AQB treatment. We performed ChIP assay to detect the H3K27me3 occupancy in the APC2 promoter region. UCSC Cancer Browser analysis revealed three H3K27me3 binding sites within the APC2 promoter (Figure S4A). The site1 was the most obviously occupied locus among the three predicted sites (Figure 4A). PCR analyses showed significantly reduced H3K27me3 occupancy and increased RNA Pol II phosphor-Ser2 occupancy (Figure 4B). Loss of EZH2 occupancy and unchanged H3 levels were observed (Figure 4C). These data indicated the transcriptional elongation of APC2 resulting from AQB treatment. We introduced HOTAIR into N33 and MDA-MB-231 cells (Figure S4B) and found that mRNA levels of APC2 were decreased after overexpression of HOTAIR, but AQB treatment abrogated HOTAIR-mediated down-regulation of APC2 (Figure 4D). These data suggested that AQB inhibits HOTAIR-mediated silencing of APC2 and restores the transcription catalyzed by RNA Pol II.

Because β-catenin is known to be a key target of APC2 31, we further examined AQB effect on β-catenin. Confocal analysis revealed decreased protein levels of β-catenin and p-β-catenin (Ser675) and increased levels of APC2 following AQB (200nM) treatment (Figure 4E). Furthermore, Western blotting showed that β-catenin and p-β-catenin levels in the nucleus and the cytosol were both reduced following AQB treatment (Figure 4F). Consistent with these findings, β-catenin downstream target genes ZEB1, SNAIL, and mesenchymal markers (N-cadherin, Vimentin) were reduced with AQB (200nM) treatment in a variety of cancer cell lines. (Figure 4G). Confocal analysis displayed the reverse of epithelial- mesenchymal transition (EMT) with decrease in filopodia, and decreased ZEB1 and Vimentin (Figure 4H). Also, cell motility was inhibited after 200nM AQB treatment in glioblastoma, breast cancer, melanoma and gastric cancer cell lines (Figure 4I). However, significantly reduced clonogenic growth was observed at the concentration of 40µM AQB in glioblastoma and breast cancer cell lines (Figure S5A) as well as cervical cancer, gastric cancer and melanoma cells which expressed elevated levels of HOTAIR and EZH2 (Figure S5B). Flow cytometry revealed that AQB (40µM) treatment elicited a S/G2 cell cycle arrest in N33 cells and a G2 arrest in MDA-MB-231 cells (Figure S5C), and induced apoptosis (Figure S5D). Taken together, these data suggest that AQB can inhibit the Wnt/β-catenin signaling pathway by upregulating APC2, leading to the reversion of EMT and proliferation suppression.

### AQB inhibits tumor cell growth and metastasis *in vivo*

**Figure 5:**
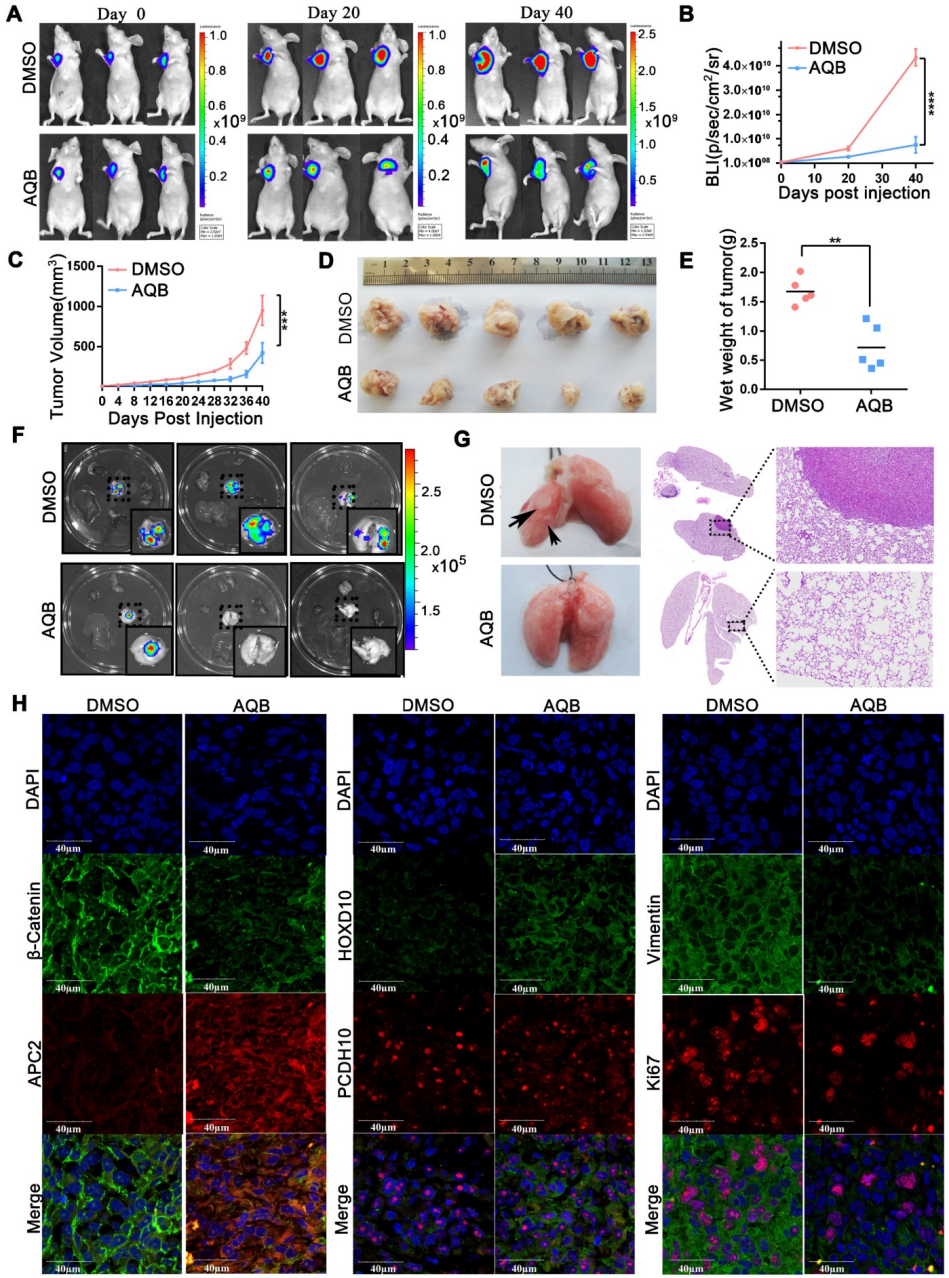
** AQB (50mg/kg) inhibits tumor growth and metastasis in MDA-MB-231 orthotopic breast cancer model.** A) Bioluminescence images from 3 representative DMSO-treated and AQB-treated mice on days 0, 20, and 40 of drug treatment. B) Quantitation of bioluminescence values for DMSO- and AQB-treated mice. C) Tumor volume was measured every 4 days after drug treatment. D) DMSO- and AQB-treated tumors were collected. E) The wet weight of DMSO-treated tumors and AQB-treated tumors. F) Bioluminescence images of the heart, liver, spleen, lung, and kidney. G) Representative lungs from DMSO- and AQB-treated mice. Arrows indicate the metastatic foci (left). Corresponding H&E staining and amplifying images (right). H) Representative confocal images display levels of APC2, β-catenin, HOXD10, PCDH10, Ki-67, and Vimentin in tumor sections. Nuclei were stained by DAPI. Scale bar, 40 µm. In B), C) and E), the data are presented as the mean±s.d.; n=5 mice. ****P<0.00001, ***P<0.0001, **P<0.001, two-tailed unpaired Student's t-test.

For *in vivo* studies, we employed an orthotopic breast cancer model by injecting MDA-MB-231 cells into the mammary fat pads of nude mice. Mice bearing tumors were treated with AQB (50mg/kg) or vehicle (DMSO) control every two days. Bioluminescence imaging and measurement of tumor volume showed that AQB treatment significantly reduced tumor growth (Figure 5A-C). At the completion of the study, orthotopic tumors and major organs were removed and examined grossly and microscopically. We found that the tumor weight in the AQB-treated group was significantly lower than the control group (Figure 5D, E). Furthermore, lung metastasis was significantly reduced in the AQB treatment group (Figure 5F, G). Immunohistochemistry analyses revealed the up-regulation of APC2, HOXD10, and PCDH10, which were HORAIR-PRC2 target genes, and down-regulation of β-catenin (Figure 5H). We also observed that AQB dramatically reduced tumor cell proliferation (Ki-67) and EMT marker Vimentin expression compared to the control group (Figure 5H). These data indicated that AQB effectively inhibits tumor growth and metastasis *in vivo*.

### Low-dose AQB enhances the toxicity of DZNep *in vitro*

**Figure 6:**
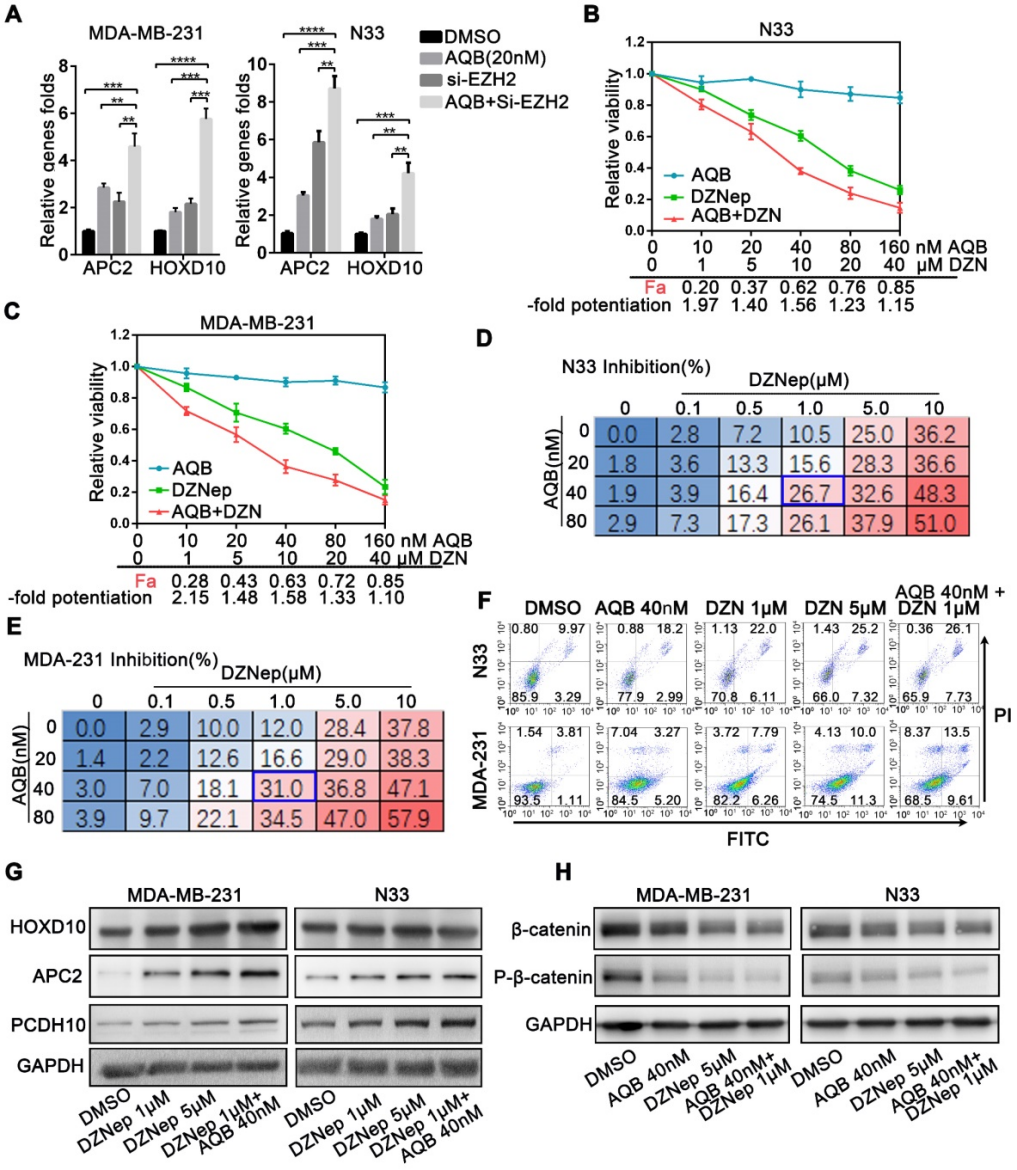
** Low dose of AQB enhances the toxicity of DZNep.** A) Cell lines were treated with 20nM AQB or siEZH2, or both for 48 hours, and then subjected to qPCR analysis of mRNA levels of APC2 and HOXD10. Data are presented as mean ± s.d.; n = 3 independent experiments. ****P<0.00001, ***P<0.0001, **P<0.001. B) and C) After treatment of N33 and MDA-MB-231 cells with different concentrations of AQB (0-160nM) and/or DZNep (0-40μM) for 48 hours, the cell viability was detected by CCK8. The fraction affected by the dose (Fa) and fold potentiation of the two drugs were calculated and listed on X axes. The -fold potentiation = Fa (combination)/Fa (DZNep). Data are presented as mean ± s.d.; n = 3. D) and E) Following treatment of cells with the indicated concentration of AQB and DZNep for 48 h, cell viability was detected by CCK-8. Data are presented as percent inhibition. The blue box indicates the optimal combination. Data are presented as mean, n = 3 independent experiments. F) After treatment with indicated drugs for 48 hours, cells were stained Annexin V-FITC and propidium iodide (PI) and subjected to flow cytometry. G) Cells were treated with indicated drugs for 48 hours, and the expression of indicated genes was determined by Western blotting. H) Immunoblotting analysis of β-catenin and p-β-catenin after treatment of cells with indicated agents for 48 hours.

Given that low dose AQB was capable of interfering with HOTAIR-EZH2 interaction to block PRC2 recruitment and increasing target genes expression, we reasoned that combination of low-dose AQB with an inhibitor of EZH2 or PRC2 could be more lethal than either one alone. To this end, we first combined AQB with EZH2 siRNA (Figure S4C) and found significantly augmented mRNA levels of HOTAIR-PRC2 target genes (Figure 6A). Therefore, we combined AQB with S-adenosylhomocysteine hydrolase inhibitor DZNep, which was the first drug proposed to indirectly inhibit EZH2 and need to be further developed 32. Interestingly, we detected strongly augmented interaction between AQB and DZNep in glioblastoma and breast cancer cells (Figure 6B, C). We further determined the inhibition rate of the combination by dose-response experiments. When combined with AQB at 40nM and DZNep at 1μM, AQB significantly enhanced the loss of cell viability caused by DZNep, and the combined inhibition rate was approximately equal to or even higher than the inhibition rate of 5μM DZNep alone (Figure 6D, E). Furthermore, the combination of AQB (40nM) and DZNep (1μM) led to more apoptosis than either agent alone (Figure 6F) and more elevated protein levels of target genes than DZNep alone (Figure 6G). Also, the combination treatment decreased β-catenin and p-β-catenin to a greater extent than either one alone potentiating the suppression of Wnt/ β-catenin signaling. (Figure 6H). These results suggested that low doses of AQB enhance the efficacy of DZNep resulting from more significant expression of HOTAIR-PRC2 target genes.

### Low-dose AQB enhances DZNep efficacy *in vivo*

We further evaluated the therapeutic efficacy of the AQB/DZNep combination in an orthotopic breast cancer animal model and a glioblastoma patient- derived xenograft (PDX) model. After construction of the tumor models, mice were randomly divided into four groups which were treated with DMSO, AQB (0.2mg/kg), DZNep (1mg/kg), and AQB (0.2mg/ kg)/DZNep (0.2mg/kg) every two days.

**Figure 7:**
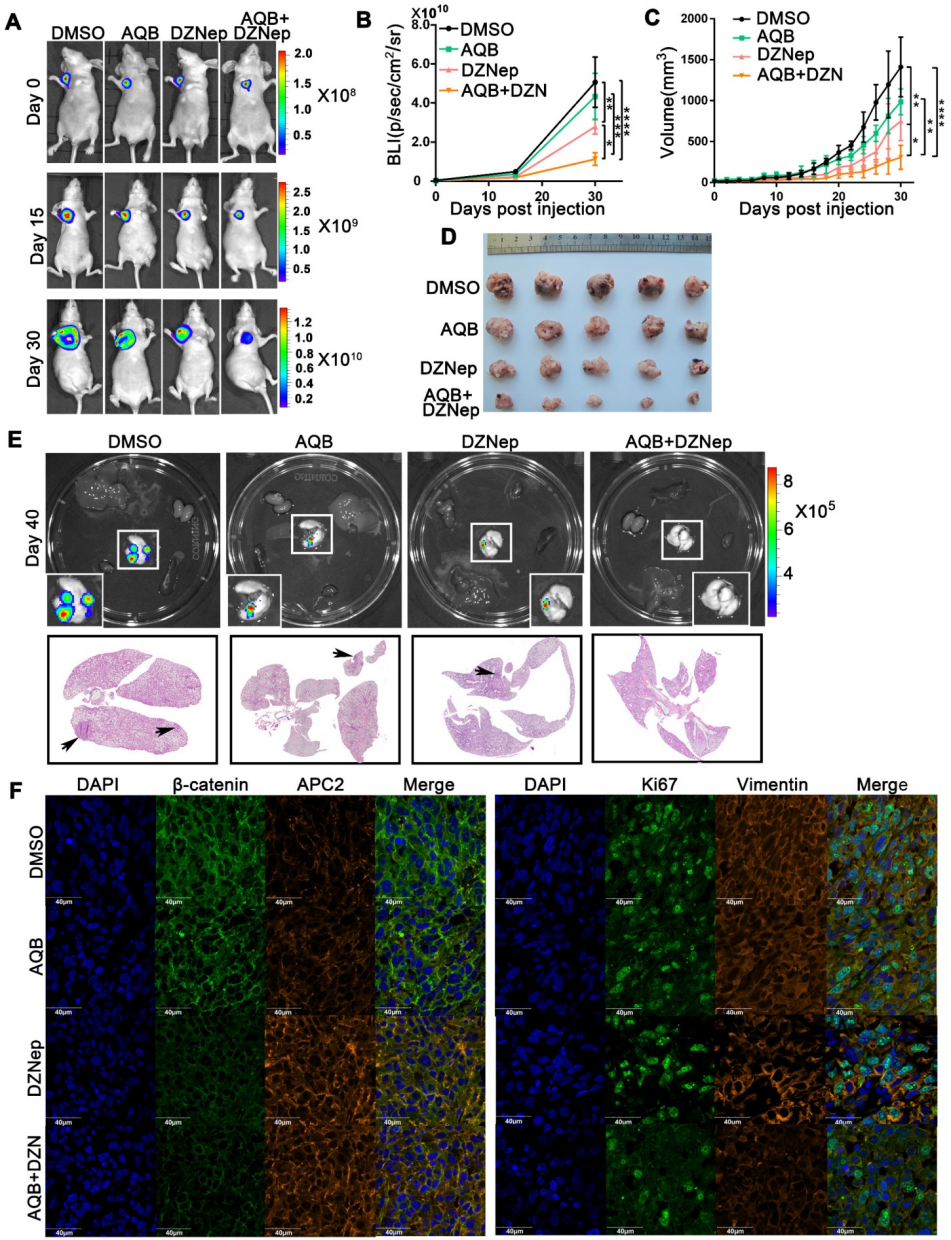
** Antitumor activity of AQB and/or DZNep in orthotopic breast cancer model.** A) Representative bioluminescence images of MDA-MB-231 orthotopic breast cancer mice that were treated with DMSO, AQB (0.2mg/kg), DZNep (1mg/kg), and the combination of AQB (0.2mg/kg) and DZNep (0.2mg/kg) at days 15 and 30. Day 0 was the time point prior to drug treatment. B) Quantitation of bioluminescence values. Data are presented as mean ± s.d.; n = 5 mice. ****P<0.00001, ***P<0.001, **P<0.001, *P<0.05. C) Tumor volume was measured every 2 days after drug treatment. Data are presented as mean ± s.d.; n = 5 mice. ****P<0.00001, **P<0.001, *P<0.05. D) Tumor size. E) Bioluminescence images of the heart, liver, spleen, lung, and kidney. Interrelated H&E staining shows the lung metastasis (bottom panel). The black arrows indicate tumors. F) Representative confocal images display levels of APC2, β-catenin, Ki-67, and Vimentin in the tumor sections. The nuclei were stained by DAPI. Scale bar, 40 µm.

In the MDA-MB-231 breast cancer model, while AQB (0.2mg/kg) group showed no significant inhibition of tumor growth when compared with the DMSO control group, the AQB/DZNep combination group displayed greater tumor growth regression than either single agent group (Figure 7A-D). At completion of the study, no lung metastasis was observed in the combination group (Figure 7E). Immunohistochemistry analyses of tumors showed an increase in APC2 and a decrease in β-catenin, Ki67 as well as Vimentin in the combination group compared with the groups treated with single agents (Figure 7F).

**Figure 8:**
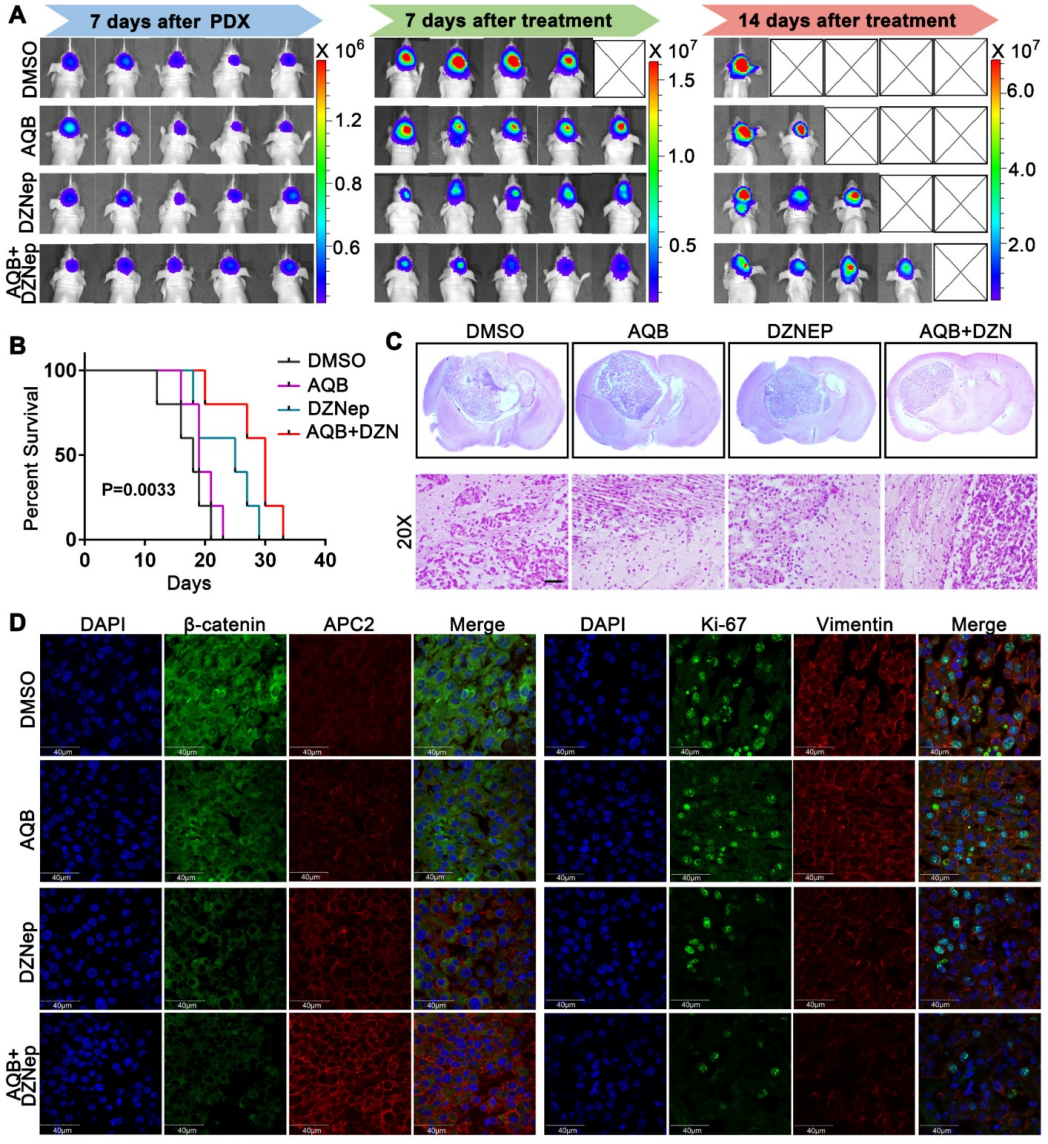
** Antitumor activity of AQB and/or DZNep in glioblastoma PDX model.** A) Bioluminescence images of glioblastoma PDX mice that were treated with DMSO, AQB (0.2mg/kg), DZNep (1mg/kg), and the combination of AQB (0.2mg/kg) and DZNep (0.2mg/kg). B) Kaplan-Meier survival plot shows the overall survival of glioblastoma PDX mice treated with regime described in A). Log-rank test was used to determine the P value. C) Representative images of HE staining of tissue sections from glioblastoma PDX mice (upper panel). The magnified (20X objective) images show the junction of the tumor and adjacent tissue (bottom panel). Scale bar, 100 µm. D) Representative confocal images of double immunostaining tumor sections with antibodies against APC2, β-catenin, Ki67, and Vimentin. Nuclei were stained by DAPI. Scale bar, 40 µm.

Patient-derived glioblastoma tissue blocks were used to generate the glioblastoma PDX model. Primary patient-derived glioblastoma cells expressed higher levels of HOTAIR and EZH2 compared with N33 cells (Figure S6A). Bioluminescence images, which were taken after treatment for 7 or 14 days, revealed much smaller tumors in the combination group than single agents alone (Figure 8A, Figure S6B). Also, the mice in the combination treatment group had more prolonged survival than mice treated with either agent alone (Figure 8B). H&E staining analysis of brain tumor sections revealed much smaller tumor size and more defined tumor boundaries in the combination group than those in control groups or those treated with either agent alone (Figure 8C). Furthermore, the tumors from the combination group expressed a much higher level of APC2 and much lower levels of β-catenin, Ki67, and Vimentin when compared to single agent and DMSO control groups (Figure 8D). These data suggested that though low dose AQB (0.2 mg/Kg) had no significant anti-tumor effect in the mouse models, the combination of AQB with DZNep (both at low doses) could potently inhibit tumor growth and metastasis providing strong evidence for the combination of AQB and DZNep as an effective strategy in the preclinical setting.

## Discussion

Several studies have demonstrated that lincRNAs are associated with PRC2 in a variety of cancer cells. Thus, lincRNAs are becoming an attractive target for the development of therapeutic strategies in human malignancies 33, 34. Recently, chemically modified oligonucleotides have been reported to successfully disrupt the interaction between lincRNA SMN-AS1 and PRC2 and be effective for the treatment of spinal muscular atrophy 22. In the present study, we identified a small- molecule compound AQB as a HOTAIR-EZH2 inhibitor by high-throughput screening. AQB selectively inhibited the interaction of HOTAIR-EZH2 which resulted in blocking the recruitment of PRC2. A recent study has demonstrated that PRC2 subunit EZH2 has the highest affinity to interact with RNA and is somewhat promiscuous 7. However, this does not exclude that lincRNAs may specifically interact with PRC2. EZH2 subunit has been shown to directly bind to HOTAIR in different models 10, 11, 13, 35. Therefore, we believed that the interaction disrupted by AQB was specific, because the association of other lincRNAs with EZH2 was not affected and the disruption of the HOTAIR-EZH2 interaction by AQB was dose-dependent with a low IC50 (42.47nM).

Loss of tumor suppressors through epigenetic silencing is a hallmark of human cancers 36. Our data demonstrated that the low dose of AQB(~40nM) was able to reverse epigenetic gene silencing mediated by HOTAIR-PRC2 through specifically disrupting HOTAIR-EZH2 interaction and blocking the recruitment of PRC2. Most of the HOTAIR-PRC2 target genes identified by ChIPseq are metastasis suppressors 14 which may explain the results that the low-dose AQB was able to suppress tumor metastasis but does not exhibit anti-proliferation effect *in vitro*. Furthermore, we found that APC2, as one of the HOTAIR-PRC2 target genes, was significantly up-regulated by AQB and led to degradation of β-catenin which resulted in the suppression of Wnt/β-catenin signaling. In this context, Wnt/β-catenin signaling has been shown to be associated with the proliferation and metastasis of tumor cells in triple negative breast cancer 37-40 and glioblastoma 19, 41. Thus, AQB-mediated suppression of the Wnt/β-catenin signaling may contribute to inhibition of tumor growth and metastasis *in vitro* and *in vivo*. Taken together, these findings provide persuasive evidence that HOTAIR-mediated modulation of cancer epigenome could be remodeled by a small compound to reverse tumor growth and metastasis affording new insights for the development of novel therapeutic approaches in clinical epigenetics.

Different tumors have different genetic background. We found that the tumor cell lines with high levels of HOTAIR and EZH2 were sensitive to AQB. Additionally, we demonstrated that HOTAIR and EZH2 are highly co-expressed in TERT Mutation Only subtype of glioma and triple-negative subtype of breast cancer. Our data indicate that AQB could benefit these subtype tumors.

DZNep has been characterized as an EZH2 inhibitor that induces the degradation of PRC2 by impairment of SAH (S-adenosyl-l-homocysteine), a global methyl group donor for diverse methyltransferases 42, 43. Therefore, DZNep does not act as a specific EZH2 antagonist. Unlike other EZH2 inhibitors, which are limited to certain hematological malignancies, DZNep has a wide range of therapeutic effects in various tumors, such as breast cancer 6, non-small cell lung cancer 44, and pancreatic cancer 45. In this study, the combination of low dose AQB with DZNep exhibited a marked anti-tumor activity superior to either agent alone in orthotopic breast cancer and glioblastoma patient-derived xenograft (PDX) models. We believe that this was largely due to the low-dose AQB blocking the PRC2 recruitment by selectively disrupting the HOTAIR-EZH2 interaction and DZNep leading to the degradation of PRC2. These distinct mechanisms of action highlight the potential advantage of the combination treatment targeting HOTAIR-EZH2-PRC2 axis. In brief, our data showed the significant enhanced anti-tumor effect of AQB and DZNep, and the combination of the two drugs at low doses elicited fewer side effects than either agent alone at a high dose.

Importantly, AQB can be easily synthesized and readily adopted for large-scale manufacturing which is a prerequisite for clinical translation. This small molecule has shown selective disruption of lincRNA- protein interaction and potent anti-tumor activity by up-regulation of tumor suppressors. Thus, lincRNA/ protein-based targeted therapy may represent an alternative and complementary strategy in lincRNA/ protein-dependent human cancers. Our study suggests AQB as a potential agent for HOTAIR-EZH2 target cancer therapy, and offers a new way to treat cancers.
