# Supplementary material for: A Compound AC1Q3QWB Selectively Disrupts HOTAIR-Mediated Recruitment of PRC2 and Enhances Cancer Therapy of DZNep
Source: Theranostics. 2019 Jun 24;9(16):4608–23. doi: 10.7150/thno.35188 (PMC6643429; doi:10.7150/thno.35188)
Supplement: Supplementary file 1 — Supplementary figures and tables. [file thnov09p4608s1.pdf]

Supplementary Materials

Supplemental Figures

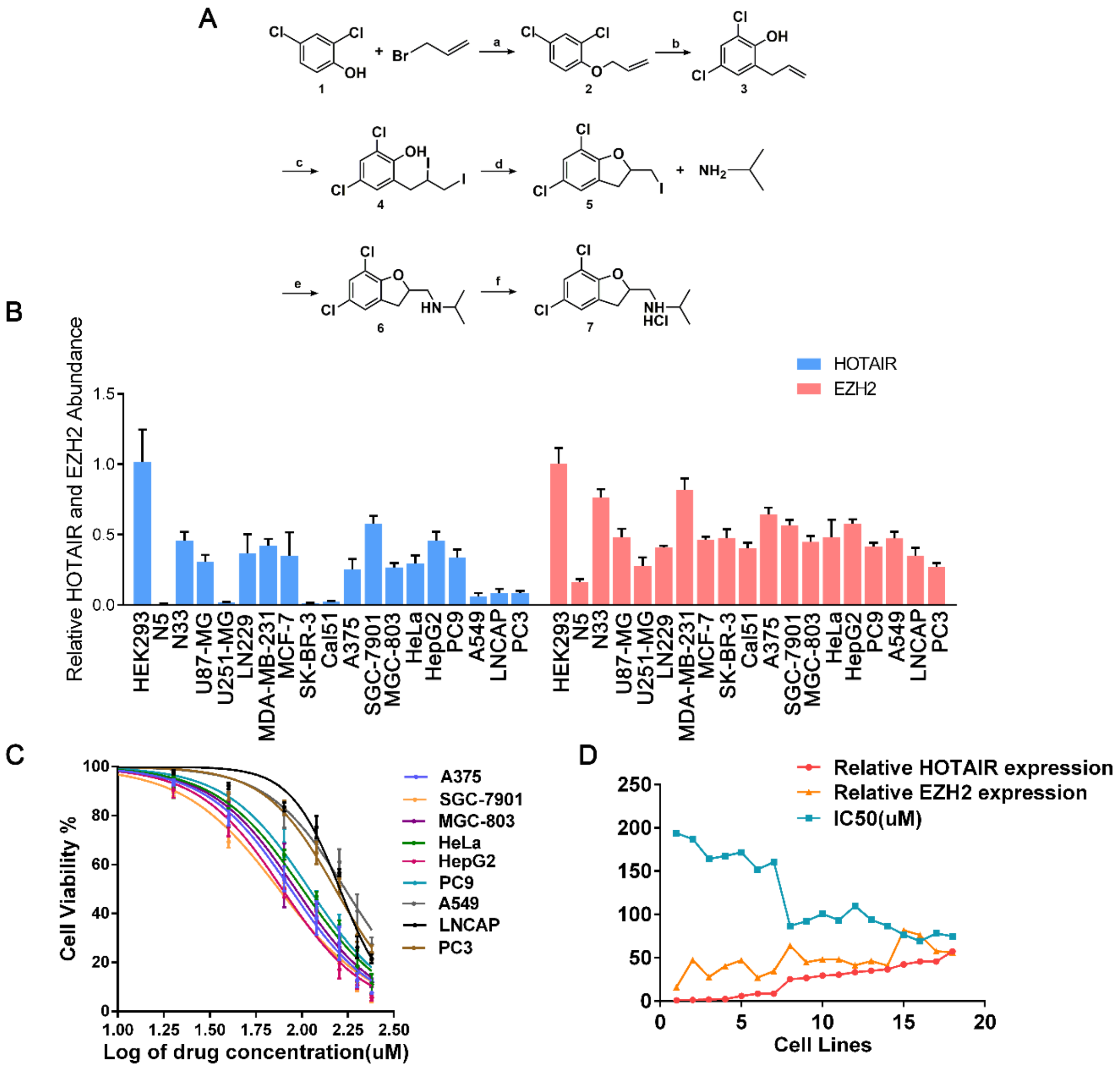

Figure S1. The expression levels of HOTAIR and EZH2 were related to AQB sensitivity in tumor cell lines. (A) Synthetic scheme of AQB; 7 is a hydrochloride translated from 6 and easy to dissolve in DMSO. Reagents and conditions: (a) K2CO3, DMF, 70°C, 2 h, then 20°C, 16 h; (b) 245-250°C, neat; (c) I2, H2O, 50°C, 12 h; (d) I2, H2O, EtOH, 50°C, 12 h then I2, SnCl4, DCM, 20°C, 16 h; (e) 40 eq isopropylamine, neat, reflux, 16 h; 40 eq isopropylamine, neat, 20°C, 16 h; 2 eq isopropylamine, DCM, 20°C, 16 h; 2 eq isopropylamine, DCM, reflux, 16 h. (B) Relative mRNA levels of HOTAIR and EZH2 by qPCR of the indicated cell lines. Values are expressed relative to the abundance in HEK293 cells. Error bars represent s.d.

(n=3). (C) Dose-response curves of AQB for 9 cell lines of 6 tumors were determined by the CCK-8 assay. Error bars represent s.d. (n=3). (D) The line chart of the correspondence between IC50 of AQB and mRNA levels of HOTAIR and EZH2 in 18 cell lines.

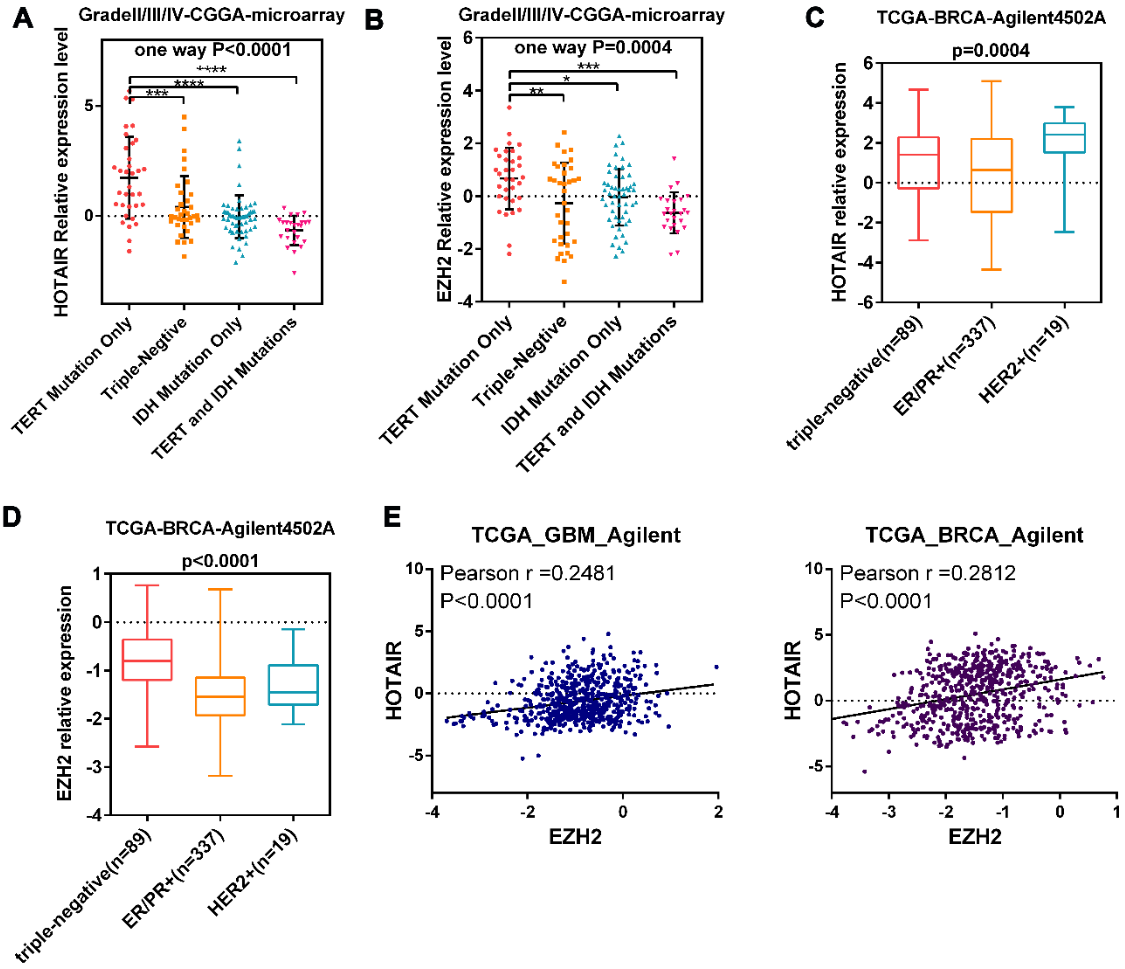

Figure S2. HOTAIR and EZH2 were highly co-expressed in the same subtype of human breast cancer and glioma. (A) (B)Analyses of HOTAIR and EZH2 expression levels in glioma subtypes using CGGA microarray data. (C) (D)Analyses of HOTAIR and EZH2 expression levels in breast cancer subtypes using TCGA data. (E) The correlation analysis between HOTAIR and EZH2 expression in TCGA databases of glioblastoma and breast cancer.

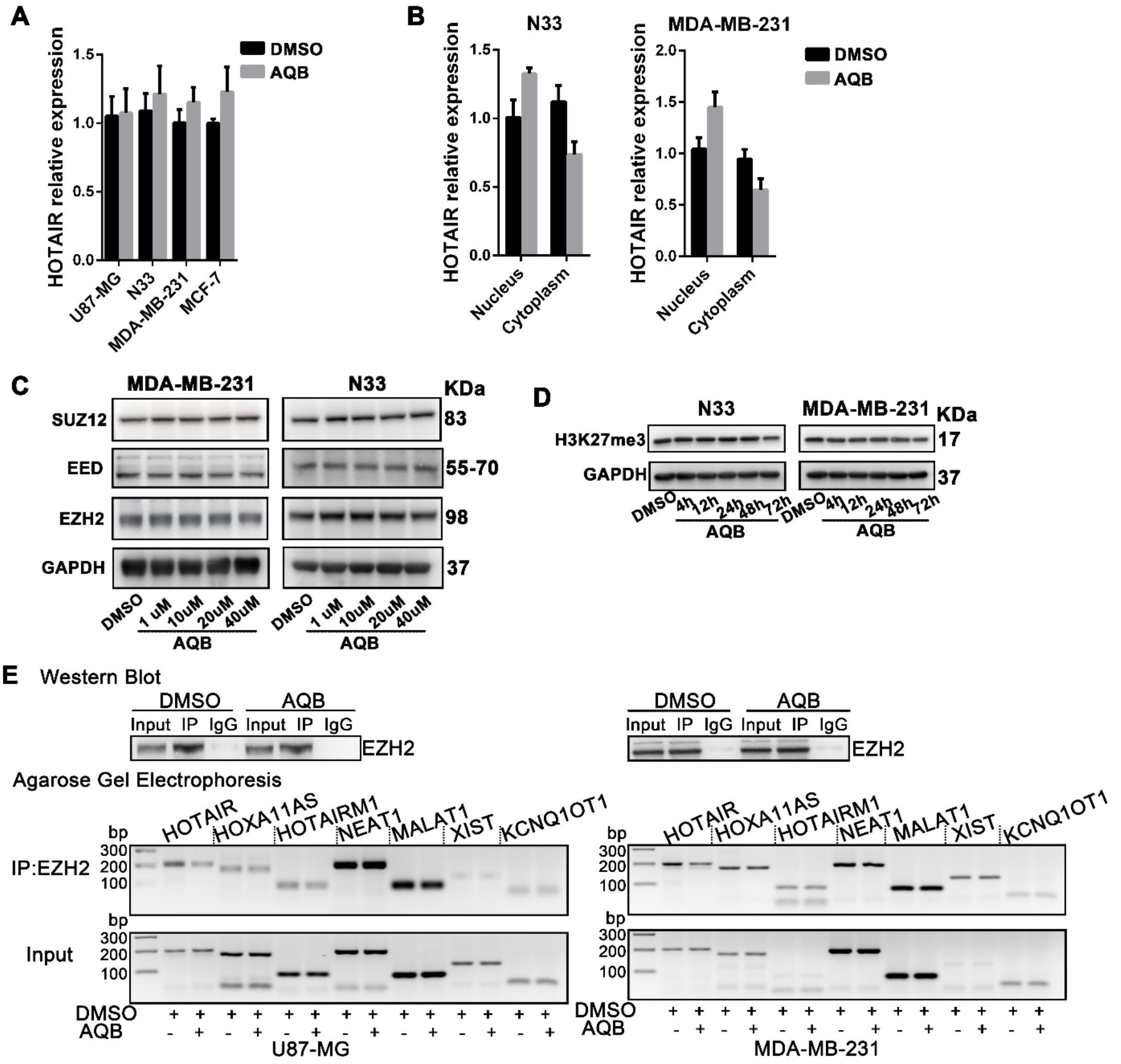

Figure S3. AQB disturbs the HOTAIR-EZH2 interaction without effect on the total level of HOTAIR and EZH2. (A) The total levels of HOTAIR in glioblastoma and breast cancer cell lines were detected by qRT-PCR after 40 μM AQB treatment for 48 hours. Data are represented as the mean±s.d.; n=3 independent experiments. No significance was found. (B) Detection of nucleoplasm levels of HOTAIR in cells treated with 40 μM AQB for 48 hours by qRT-PCR. Data are represented as the mean±s.d.; n = 3 independent experiments. No significance was found. (C) Western blot analysis of EZH2, EED and SUZ12 in cells treated with the indicated concentrations of AQB for 48 hours. (D) Western blot analysis of global levels of H3K27me3 in cells treated with 40 μM AQB for the indicated period. (E) 50μL beads suspension was removed during the last wash and was used to test the efficiency of

EZH2-immunoprecipitation by Western blotting. IgG was the negative control in RIP assay. Agarose gel electrophoresis experiment was employed to examine immunoprecipitated RNA levels after the treatment with AQB at dose 40μM for 48 hours. Input was used as a loading control.

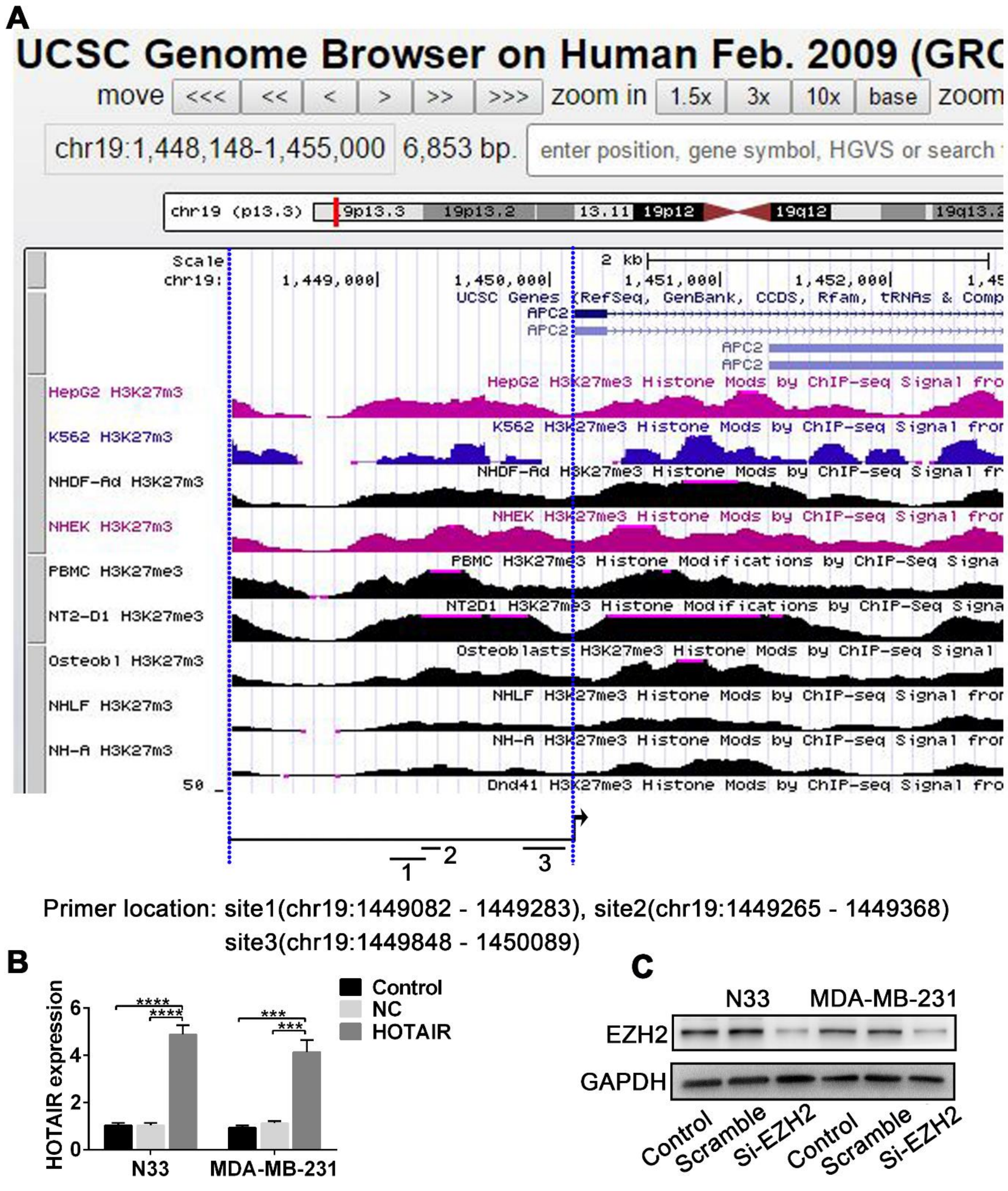

Figure S4. Prediction of H3K27me3 binding sites of APC2 and the efficiency detection of HOTAIR and siEZH2 transfection. (A) H3K27me3 binding sites of APC2 and location of the three primers predicted by the UCSC Genome Browser. (B) Detection of HOTAIR transfection efficiency by RT-qPCR. Data are represented as the mean±s.d.; n=3 independent experiments. ****P<0.00001, ***P<0.0001, two-tailed unpaired Student's t-test. (C) Detection of siEZH2 transfection efficiency by Western blot analysis.

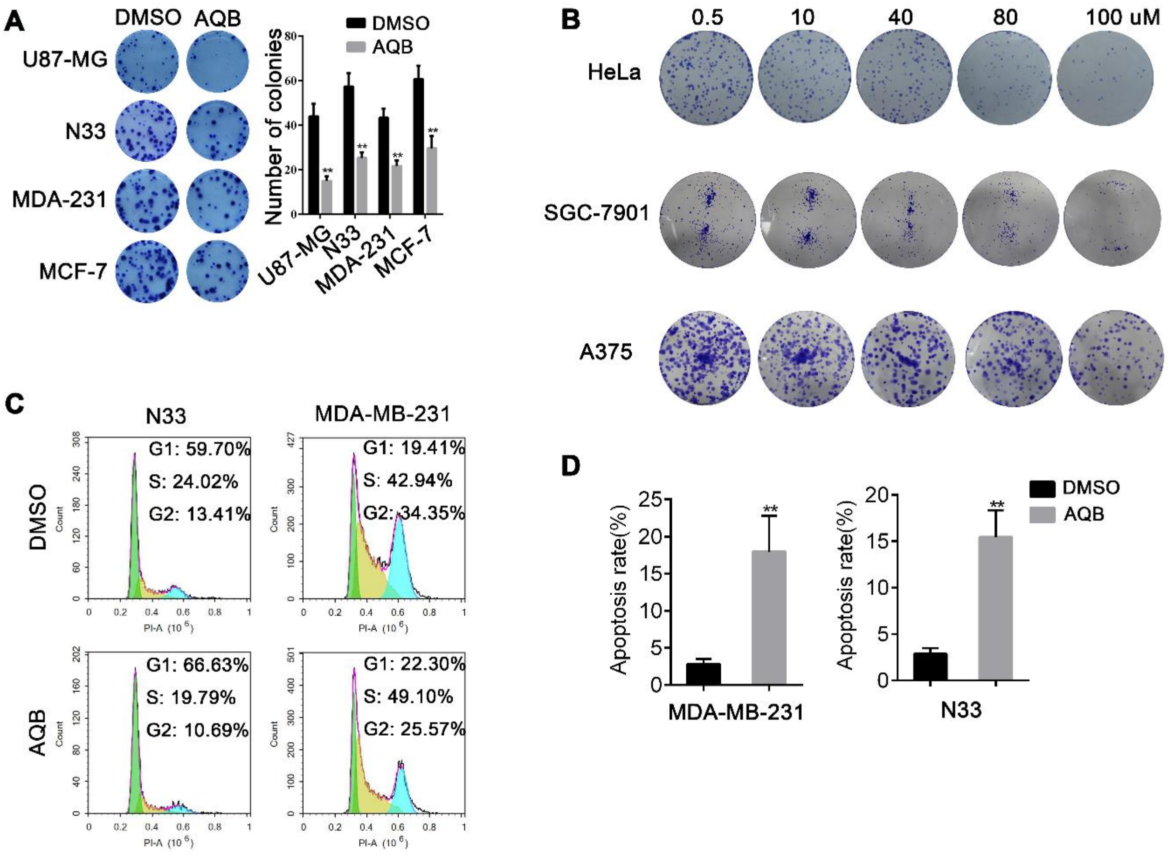

Figure S5. The AQB inhibited tumor cells proliferation and induced apoptosis. (A) Clonogenic assays were performed in glioblastoma and breast cancer cell lines after treatment with 40 µM AQB. The column chart showed the number of clones. Data are represented as the mean±s.d.; n=3. **P<0.001, two-tailed unpaired Student's t-test. (B) Clonogenic assays were performed in cervical cancer, gastric cancer, and melanoma cell lines after treatment with the indicated concentrations of AQB. (C) Flow cytometry was performed to detect the G1/S arrest in glioblastoma and breast cancer cell lines after treatment with 40 µM AQB for

48 hours. (D) Apoptosis detection after 48 hours of treatment with 40 µM AQB. Data are represented as the mean±s.d.; n=3. **P<0.001, two-tailed unpaired Student's t-test.

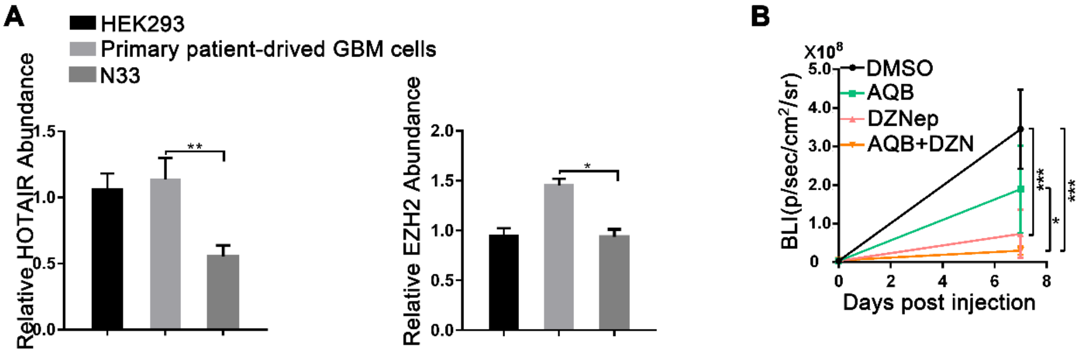

Figure S6. Antitumor activity of AQB and/or DZNep in glioblastoma PDX model. (A) Relative mRNA levels of HOTAIR and EZH2 by qRT-PCR of the primary patient-derived glioblastoma cells and N33 cells. Values are expressed relative to the abundance in HEK293 cells. Error bars represent s.d. (n=3), **P<0.01, *P<0.05. (B) Quantitation of bioluminescence values for DMSO-, 36806-, DZNep-, and combination-treated mice. Data are represented as the mean ± s.d.; n=5 mice. ***P<0.001, *P<0.05, one-way ANOVA followed by Bonferroni multiple comparison tests.

Supplemental Tables

Table S1. RT-qPCR primers.

Primers of lncRNAs.

HOTAIR-F	ATAGGCAAATGTCAGAGGGTT
HOTAIR-R	TCTTAAATTGGGCTGGGTC
HOXA11-AS-F	CCCTCATCTTCCCTGCCTACCT
HOXA11-AS-R	GGTTTCCCGAGTCCTCAGCT
HOTAIRM1-F	TTGCCTGAACCCATCAACAG
HOTAIRM2-R	CACCCACATTTCAACCCCCT
NEAT1- F	CCAGTTTTCCGAGAACCAAA
NEAT1-R	ATGCTGATCTGCTGCGTATG
MALAT1 -F	GACGGAGGTTGAGATGAAGC

MALAT1 -R	ATTCGGGGCTCTGTAGTCCT
XIST-F	CTTAAAGCGCTGCAATTCGCT
XIST-R	AGGGTGTTGGGGGACTAGAA
KCNQ1OT1-F	TTGGATTACTTCGGTGGGCT
KCNQ1OT1-R	ACACGGATGAAAACCACGCT
GTL2-F	CTCCTTGCATCAGGTAGGGG
GTL2-R	CACGTGCCTTTGTGAGCGT
H19-F	ATGACTGGGACCCAAGGACT
H19-R	ATGTCCTGCTTGTCACGTCC

Primers of target genes.

PCDH10-F	CCCGTCTACACTGTGTCCCT
PCDH10-R	GGAGTACACGACCTCACCGT
PCDHB5-F	AGGTGTGTTTGACCGGAGAC
PCDHB5-R	TCCCTATTTCTTCACCAGCG
HOXD10-F	CCGACAGGCAGGTCAAGATT
HOXD10-R	TTTCCGCTTTCCCAGTCCTC
APC2-F	CAGGAGCTGAAGATGGCGAG
APC2-R	GGTGCTTCAGGACCTCCTTC
NLK-F	TGATCACGGATCTGTTGGGC
NLK-R	ACCAACATCCTGCAAAGGAGAT

Primers of GAPDH and EZH2.

GAPDH-F	GGTGGTCTCCTCTGACTTCAACA
GAPDH-R	GTTGCTGTAGCCAAATTCGTTGT
EZH2-R	GGACTCAGAAGGCAGTGGAG
EZH2-F	CTTGAGCTGTCTCAGTCGCA

ChIP primers of APC2.

APC2-site1-F	CAAATCCAGACGCAGGAGGT
APC2-site1-R	GGTGAGACAGCCAATGAGGG
APC2-site2-F	GTGCTGTGAGTCTGTGTCCA
APC2-site2-R	GGATGCTCCATGAGTCCCTG
APC2-site3-F	GGGGCCTTGAATACTCGGAC
APC2-site3-R	GGACACAGACTCACAGCACA

Table S2. Sequences of siEZH2 and HOTAIR-probes.

Sequences of biotin probes in ChIRP.

HOTAIR-1	TGTGGAAGCTTTCGGATCAA
HOTAIR-2	TTAGGGACCTGAGGGTCTAA
HOTAIR-3	AAATCCGTTCCATTCCACTG
HOTAIR-4	AATAAAGACGCCCCTCCTTC
HOTAIR-5	TTTCAGCCTTTTCTCTGCCA
HOTAIR-6	GGTGTAATTGCTGGTTTAGG
HOTAIR-7	TAAACCTCTGTCTGTGAGTG
HOTAIR-8	AGGTTTTTCCAGCGTTCTCT
HOTAIR-9	ATTAATTAGCGCCTCCCAGT

HOTAIR-10	CTGTTTGGGCCTCCTAAAAT
HOTAIR-11	TGTTCCTCTCAAATTCCGGA
HOTAIR-12	TGTGCTGCCAGTTAGAAAAG
HOTAIR-13	CTGTGTCTACATGCATCACT
HOTAIR-14	CATACCTACCCAATGTATGG
Mock	CCTCTTACCTCAGTTACAATTTATA

Sequences of siEZH2.

siEZH2	ACUCUGAAUGCAGUUGCUTT
Scramble for siEZH2	AGCAACUGCAUUCAGAGUTT
